# Stress-induced release of the S100A8/A9 alarmin is elevated in coronary artery disease patients with impaired cortisol response

**DOI:** 10.1038/s41598-017-17586-6

**Published:** 2017-12-13

**Authors:** L. Jonasson, H. Grauen Larsen, A. K. Lundberg, B. Gullstrand, A. A. Bengtsson, A. Schiopu

**Affiliations:** 1 Division of Cardiovascular Medicine, Department of Medical and Health Sciences, Linköping University, Linköping, Sweden; 2 Experimental Cardiovascular Research Unit, Department of Clinical Sciences Malmö, Lund University, Lund, Sweden; 3 Department of Cardiology, Skane University Hospital Malmö, Malmö, Sweden; 4 Section of Rheumatology, Department of Clinical Sciences Lund, Lund University and Skane University Hospital Lund, Lund, Sweden

## Abstract

Psychological stress is thought to be an important trigger of cardiovascular events, yet the involved pathways and mediators are largely unknown. Elevated systemic levels of the pro-inflammatory alarmin S100A8/A9 correlate with poor prognosis in coronary artery disease (CAD) patients. Here, we investigated the links between S100A8/A9 release and parameters of anti-inflammatory glucocorticoid secretion in two different cohorts subjected to a psychological stress test. In the first cohort of 60 CAD patients, psychological stress induced a rapid increase of circulating S100A8/A9. This rapid S100A8/A9 response strongly correlated with elevated evening saliva cortisol levels, suggesting an association with a dysregulated hypothalamic–pituitary–adrenal (HPA) axis. In the second cohort of 27 CAD patients and 28 controls, elevated S100A8/A9 levels were still detectable 24 h after stress in 40% of patients and 36% of controls, with a tendency for higher levels in patients. The sustained S100A8/A9 response was associated with a poor rapid cortisol release after stress in patients, but not in the control group. Our findings reveal for the first time that acute psychological stress induces elevated levels of S100A8/A9. We also provide hypothesis-generating evidence that dysregulated cortisol secretion in CAD patients might be associated with an exaggerated pro-inflammatory S100A8/A9 response.

## Introduction

Inflammation plays a central role in the development of cardiovascular disease (CVD). Immune and inflammatory cells mediate atherogenesis and plaque destabilization, and the circulating levels of inflammatory mediators have been linked with disease progression and prognosis^1^. The S100 proteins A8 and A9 are alarmins belonging to the S100 calcium-binding protein family. They are expressed in neutrophils, monocytes, thrombocytes and dendritic cells but also in activated macrophages, vascular endothelial cells and fibroblasts^2,3^. S100A8 and S100A9 exist as homodimers, but preferably form the heterodimer S100A8/A9, also known as calprotectin. S100A8/A9 constitutes approximately 45% of all cytosolic proteins in neutrophils and about 1% in monocytes^4^.

S100A8/A9 is an endogenous ligand of toll-like receptor 4 (TLR4)^5,6^ and of the receptor for advanced glycation end-products (RAGE)^7–9^, present on various cell types. S100A8/A9 binding to these receptors promotes the synthesis of pro-inflammatory mediators and has been shown to be involved in the pathogenesis of acute coronary syndrome (ACS)^2^. Systemic levels of S100A8/A9 correlate with established markers of systemic inflammation in patients with stable coronary artery disease (CAD)^10^, and with the severity of CAD in type 1 and type 2 diabetic patients^2^. Further, S100A8/A9 is elevated in vulnerable human carotid plaques^11^ and systemic levels are associated with the long-term risk of CV events and CV death in middle-aged individuals without previous CVD^12^. In ACS patients, S100A8/A9 levels are increased in blood and at the site of coronary occlusion, and recovered thrombi from coronary vessels contain S100A8/A9 positive cells^13^. These findings suggest that S100A8/A9 is released locally from the ischemic myocardium and diffuses into the systemic circulation. Moreover, the magnitude of S100A8/A9 response has prognostic value, as ACS patients with persistently elevated levels of S100A8/A9 30 days after the acute event have a significantly higher risk to suffer recurrent CV events^14^.

Acute psychological stress triggers the release of inflammatory mediators in humans^15,16^ and may thereby increase the risk for CV disease^17,18^. Glucocorticoids regulate immune and inflammatory pathways, and are essential for dampening the inflammatory response associated with acute stress^19,20^. Chronic inflammation in pathological conditions, such as autoimmune diseases and depression, has been linked to impaired HPA axis function^21^. This is also proposed as a potential mechanism for low-grade chronic inflammation in CAD^22^. Compared to healthy controls, CAD patients have a flat diurnal cortisol rhythm and a blunted cortisol response to acute stress, a cortisol pattern that is associated with elevated levels of pro-inflammatory markers^23^. An imbalance between pro- and anti-inflammatory pathways in CAD patients is generally believed to promote disease progression and lead to worse clinical outcome^24^. Laboratory psychological stress tests may therefore be useful to reveal whether such an inflammatory imbalance exists.

S100A8/A9 is readily produced and stored in neutrophils and quickly released upon activation, serving as an ideal biomarker for monitoring rapid inflammatory responses. However, it has not previously been studied whether acute psychological stress triggers S100A8/A9 release. Here, we investigated the S100A8/A9 response to acute psychological stress in two different cohorts of CAD patients and related the findings to cortisol reactivity as well as to diurnal cortisol rhythm. We hypothesized that S100A8/A9 release is an integral part of the inflammatory response to stress, and that this response may be exacerbated in CAD patients with a dysregulated cortisol metabolism.

## Results

### Rapid S100A8/A9 response to acute psychological stress

**Figure 1:**
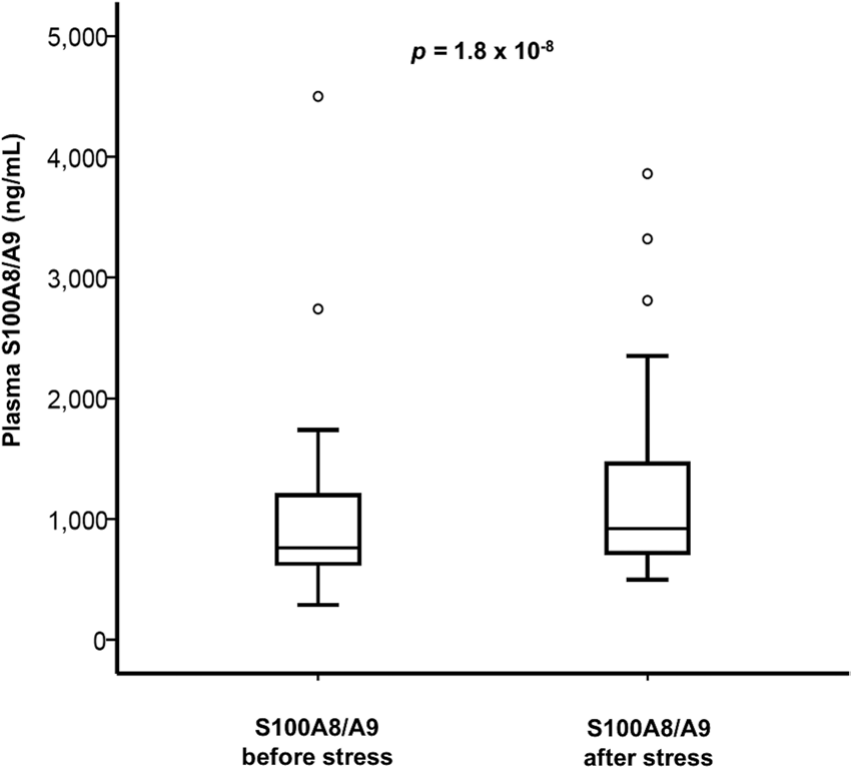
Circulating S100A8/A9 before and after acute psychological stress (Study I). Plasma S100A8/A9 increased significantly in CAD patients (n = 60) subjected to acute psychological stress. Plasma samples were collected before and at 20 minutes after the completion of the psychological stress test. The difference between the groups was calculated using the paired Wilcoxon Signed Ranks test. °Denotes outliers.

**Figure 2:**
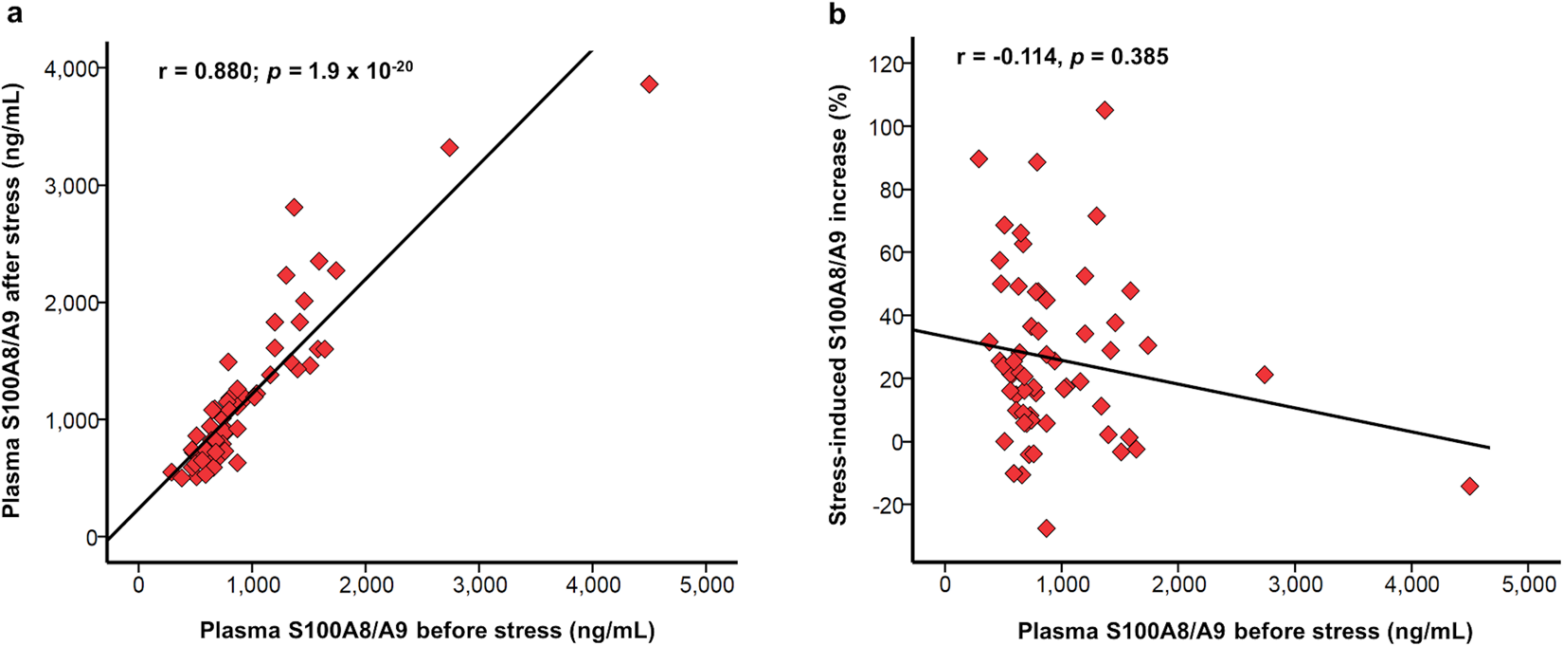
Correlation between baseline S100A8/A9 and the rapid S100A8/A9 response induced by acute psychological stress (Study I). (**a**) Correlation between S100A8/A9 in plasma before and 20 minutes after the end of the psychological stress test. (**b**) Correlation between S100A8/A9 in plasma before the test and the percentage S100A8/A9 increase compared to baseline induced by acute psychological stress. The correlations were examined using the Spearman test.

In Study I, we measured the early changes in systemic S100A8/A9 levels induced by acute psychological stress in 60 CAD patients. The clinical and biochemical characteristics of the study group are presented in Table 1. Psychological stress induced a significant elevation in plasma S100A8/A9 measured at 20 min after test completion compared to baseline [median (IQR) 750 (615–1190) ng/mL pre-stress vs 910 (720–1452) ng/mL post-stress; *p* = 1.8 × 10^−8^] (Fig. 1). There was a strong positive association between baseline and post-stress S100A8/A9 levels (*r* = 0.880, *p* = 1.9 × 10^−20^) (Fig. 2a), and both correlated positively with blood neutrophil counts (*r* = 0.301, *p* = 0.019 and *r* = 0.302, *p* = 0.019, respectively). However, the relative S100A8/A9 increase expressed as percent of baseline levels did not correlate with pre-stress S100A8/A9 (*r* = −0.114, *p* = 0.385) (Fig. 2b) or with the number of circulating neutrophils (Table 2), suggesting that the individual reactivity to psychological stress was independent of the habitual S100A8/A9 levels and of the neutrophil counts in blood. We found a positive correlation between the relative S100A8/A9 increase and systolic blood pressure at 10 minutes after the test (*r* = 0.287, *p* = 0.016), suggesting that individuals with a stronger S100A8/A9 response had an impaired systolic blood pressure recovery after the stressor. There were no correlations between the S100A8/A9 response and diastolic blood pressure or heart rate at any time point.

**Table 1:** Clinical and biochemical parameters of the CAD patients and controls included in studies I and II

	CAD patients Study I (N = 60)	CAD patients Study II (N = 27)	Controls Study II (N = 28)	P^*^ Study II
Age (years)	65.1 (8.9)	61 (6.0)	61 (6.0)	n.s.
Male sex, n (%)	51 (85.0)	22 (81.4)	23 (82.1)	n.s.
Smoking, n (%)	5 (8.3)	13 (48.1)	9 (32.1)	n.s.
Diabetes, n (%)	11 (18.3)	1 (3.7)	1 (3.6)	n.s.
Systolic blood pressure (mmHg)	138 (15)	136 (21)	144 (16)	n.s.
Diastolic blood pressure (mmHg)	79 (9)	80 (10)	85 (8)	0.012
Heart rate (beats/min)	60 (10)	64 (11)	71 (13)	0.041
BMI (kg/m^2^)	27.4 (3.3)	27 (3)	27 (3)	n.s.
Plasma lipids (mmoL/L)				
Total cholesterol	3.76 (0.78)	4.44 (0.78)	5.89 (0.92)	6.9 × 10^−8^
LDL	1.99 (0.55)	2.40 (0.63)	3.80 (0.92)	4.6 × 10^−8^
HDL	1.17 (0.35)	1.27 (0.33)	1.38 (0.31)	n.s.
TG	1.39 (0.70)	1.72 (0.75)	1.75 (0.91)	n.s.
Circulating cell populations (million/dL)				
Leukocytes	6.6 (2.1)	6.4 (1.3)	6.6 (1.5)	n.s.
Neutrophils	3.7 (1.4)	3.7 (1.1)	3.7 (1.1)	n.s.
Monocytes	0.5 (0.2)	0.7 (0.3)	0.5 (0.2)	n.s.
Lymphocytes	2.1 (0.9)	1.9 (0.6)	2.2 (0.6)	n.s.
Medication				
Statin, n (%)	59 (98.3)	25 (92.6)	3 (10.7)	3.3 × 10^−10^
ACE/ARB, n (%)	42 (70.0)	13 (48.1)	3 (10.7)	0.003
Betablocker, n (%)	46 (76.7)	24 (88.9)	5 (17.9)	9.9 × 10^−8^
Calcium channel blockers, n (%)	21 (35.0)	4 (14.8)	3 (10.7)	n.s.

Continuous variables are presented as mean (SD).

^*^Comparison between CAD patients and controls in Study II. Student’s T-test was used for continuous variables. The chi-square test or Fischer’s exact test were used to compare categorical variables, as appropriate.

**Table 2:** Potential determinants of the rapid S100A8/A9 response induced by acute psychological stress in CAD patients (Study I).

	Spearman correlation		Multivariable linear regression^*^	
	r	P	Beta coefficient	P
Age	0.029	n.s.	−0.100	n.s.
Sex	—	—	0.098	n.s.
BMI	−0.148	n.s.	−0.274	n.s.
Diabetes	—	—	0.068	n.s.
Hypertension	—	—	−0.138	n.s.
Smoking	—	—	−0.029	n.s.
Plasma lipids				
Total cholesterol	−0.033	n.s.	—	—
LDL	−0.087	n.s.	−0.211	n.s.
HDL	−0.061	n.s.	−0.124	n.s.
TG	−0.100	n.s.	−0.050	n.s.
Circulating cell populations				
Leukocytes	0.113	n.s.	—	—
Neutrophils	0.154	n.s.	−0.106	n.s.
Monocytes	0.048	n.s.	0.041	n.s.
Lymphocytes	0.011	n.s.	−0.101	n.s.
Saliva cortisol				
Morning	−0.006	n.s.	0.165	n.s.
Evening	0.315	0.016	0.635	0.004
Cortisol response to stress^#^	0.032	n.s.	0.218	n.s.
Medication				
Statin	—	—	−0.049	n.s.
Betablocker	—	—	0.082	n.s.
ACE/ARB	—	—	0.205	n.s.
Calcium-channel blockers	—	—	0.038	n.s.

^*^Multivariable linear regression with stress-induced S100A8/A9 increase, expressed as percentage of baseline, as dependent variable.

^#^Percent cortisol increase at 20 minutes after the stress test compared to baseline.

Further, we assessed whether the rapid stress-induced increase of S100A8/A9 in CAD patients was associated with the presence of CV risk factors, blood immune cell counts and parameters of cortisol homeostasis (Table 2). In a bivariate Spearman correlation analysis, the relative S100A8/A9 increase was not associated with any of the considered CV risk factors or cell counts, and did not correlate with the rapid cortisol response to psychological stress. However, the S100A8/A9 release strongly correlated with evening saliva cortisol (Table 2). After adjustment for age, sex, BMI, diabetes, hypertension, smoking, plasma lipids, blood immune cell counts, morning saliva cortisol, cortisol response to the stress test and medication in a multivariable linear regression analysis, evening saliva cortisol remained a potent determinant of stress-induced S100A8/A9 increase (Table 2).

### Sustained S100A8/A9 response to acute psychological stress

In Study II, we measured the changes in systemic S100A8/A9 levels 24 hours after the psychological stress test in 27 CAD patients and 28 healthy controls. The clinical and biochemical characteristics of the participants, previously described by Nijm *et al*.^23^ are shown in Table 1. The patients had significantly lower levels of total cholesterol and LDL cholesterol, as well as lower diastolic blood pressure and heart rate compared to controls, likely due to medication. However, both patients and controls reacted to the psychological stress test with significant increases in blood pressure and heart rate, as we have previously described in this cohort^23^. When all subjects were considered, there were no significant differences between patients and controls regarding the stress-induced changes in S100A8/A9 levels relative to baseline [median (IQR) 7 (−34–106)% vs 33 (−19–62)%, *p* = 0.960]. However, there was a large variability in the relative change in S100A8/A9 from baseline to 24 h within both groups, indicating that the S100A8/A9 response to stress was highly heterogeneous among individuals. A sustained S100A8/A9 response, defined as an increase of at least 50% at 24 h compared to baseline, was seen in 11 (40%) of the patients and 10 (36%) of the controls. Among these subjects, patients tended to have a slightly stronger S100A8/A9 response compared to controls [median (IQR) 155 (88–268)% vs 69 (57–205)*%, p* = 0.049].

**Figure 3:**
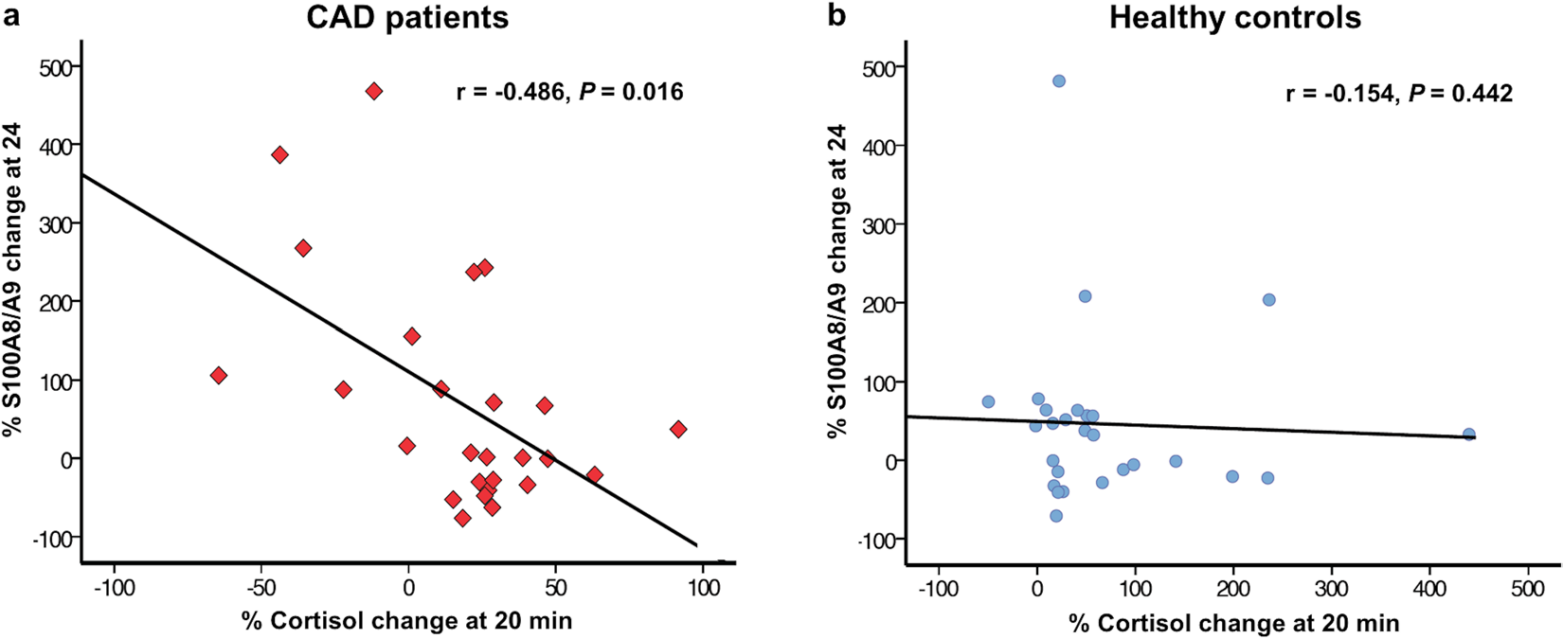
Correlation between the rapid cortisol response to psychological stress and the sustained S100A8/A9 response at 24 hours after the test (Study II). Correlation between the rapid cortisol response measured in saliva at 20 min after the psychological stress test and the S100A8/A9 increase at 24 hours after the stress test, expressed as percentage increase relative to baseline, in CAD patients (**a**) and healthy controls (**b**). The correlations were examined using the Spearman test.

The acute cortisol response to stress, defined as percentage cortisol increase at 20 minutes after the test relative to baseline values, was significantly lower in CAD patients compared to controls [median (IQR) 25 (0.7–31)% vs. 41 (17–88)%, *p* = 0.029]. The variation in cortisol levels correlated negatively with the S100A8/A9 response at 24 h after stress in CAD patients (*r* = −0.486, *p* = 0.016) (Fig. 3a), but no such correlation was present in healthy controls (*r* = −0.154, *p* = 0.442) (Fig. 3b). The data suggest that CAD patients with a poor cortisol reaction to acute psychological stress maintain a pro-inflammatory status characterized by elevated levels of S100A8/A9 that persist for at least 24 hours after the stressor.

## Discussion

In this study we evaluated for the first time the stress-induced response of the pro-inflammatory alarmin S100A8/A9 in humans. We found that acute psychological stress induced a rapid S100A8/A9 increase in a population of CAD patients, already detectable at 20 minutes after the end of the stressor. In a second cohort of CAD patients and healthy controls, elevated S100A8/A9 levels were still present 24 h after stress in a substantial fraction of participants from both groups, indicating that the release of S100A8/A9 in response to stress is not only restricted to CAD patients. Among individuals with a sustained S100A8/A9 increase at 24 h after stress, there was a slight tendency for a higher increase in patients compared with controls, but the S100A8/A9 response was highly heterogeneous within each group. Interestingly, the magnitude of S100A8/A9 rise at 24 h correlated inversely with the ability of CAD patients to mount an adequate rapid cortisol response to stress, while no such correlation was seen in controls.

Psychological stress, both chronic and acute, has long been identified as a risk factor for CVD^18^. The underlying mechanisms are not completely understood but several studies have shown that stress causes potent inflammatory reactions in peripheral blood mononuclear cells and elevated levels of pro-inflammatory cytokines^15,22,25^. CAD patients are often characterized by the presence of low-grade systemic inflammation and it has also been reported that they have a more potent increase of inflammatory mediators in response to stress compared to healthy individuals^23,26^. Additionally, patients who had reported emotional distress immediately before the onset of myocardial infarction were characterized by a significant increase of monocyte-platelet and neutrophil-platelet aggregation in response to a mental stress test, compared with patients who had not reported any emotional trigger^27^. The patients with an identifiable emotional trigger also presented delayed recovery of systolic blood pressure after stress^27^, which is in line with our findings of a correlation between a strong S100A8/A9 response and elevated blood pressure levels immediately after stress.

S100A8/A9 is a biomarker of innate immune activation and an active mediator in the pathogenesis of CVD^2^. Experimental and clinical studies have identified S100A8/A9 as an important pro-atherogenic factor^11,28,29^, and elevated S100A8/A9 levels have been correlated with increased risk for coronary events and mortality in healthy middle-aged individuals^12^. In myocardial infarction patients, S100A8/A9 is elevated both systemically and at the site of coronary occlusion^13^ and high S100A8/A9 levels correlate with poor prognosis^14^. In experimental studies, S100A8/A9 has been shown to promote cardiomyocyte dysfunction and post-ischemic heart failure^9,30^. Moreover, S100A8/A9 expression induced by angiotensin II in myocardial fibroblasts induces cardiac inflammation, fibrosis, hypertrophy and dysfunction^31^. Taken together, the data indicate that elevated S100A8/A9 levels contribute to accelerated CAD development and a worse prognosis once an acute coronary event has occurred. Thus, it is reasonable to speculate that CAD patients with repeated and sustained elevations of S100A8/A9 in response to daily stressors are at higher risk to suffer recurrent coronary events and heart failure. Adequately powered studies, designed for long-term follow-up of CAD patients, are required to address this hypothesis.

Interestingly, we found no correlation between the rapid S100A8/A9 response to psychological stress and the baseline S100A8/A9 concentration or blood neutrophil counts. It is unlikely that the rapid S100A8/A9 increase detected at 20 minutes after the stress test is due to a fast rise in circulating neutrophil numbers within this short time frame. Instead, we speculate that the magnitude of S100A8/A9 increase is determined by neutrophil responsiveness, i.e. the ability of neutrophils to become activated and release S100A8/A9 in response to psychological stress. In support of this hypothesis, previous studies have shown that neutrophils from CAD patients are more prone to *ex-vivo* stimulation compared to neutrophils from controls^32^. However, we cannot exclude the contribution of other cell types to the rise in S100A8/A9 observed in our study.

In Study I, we found a significant relationship between the rapid release of S100A8/A9 in response to stress and evening cortisol levels. Cortisol regulates the secretion of inflammatory mediators by inhibiting pro-inflammatory cytokine production and by stimulating the production of anti-inflammatory cytokines in leukocytes^33–35^. In line with this, S100A8/A9 has been shown to be negatively regulated by glucocorticoids^36^. Cortisol reactivity is a vital adaptive physiological mechanism that enables the organism to cope with stressful situations^19^. A flat cortisol diurnal slope due to high evening levels is generally considered to be indicative of long-term overstimulation of the HPA axis, which gradually becomes hypo-reactive in response to acute stimuli^37^. A flat diurnal cortisol slope has been associated with a higher prevalence of coronary calcifications^38^, and with increased risk of coronary events^39^. Moreover, in a large population-based study, high evening cortisol levels and a flatter diurnal cortisol slope were clearly linked to increased risk for cardiovascular disease mortality, independently of traditional risk factors^40^. In patients with established CVD, a flat diurnal cortisol slope has been shown to predict worse outcome after coronary by-pass surgery^41^, and elevated evening cortisol has also been associated with increased mortality in patients with systolic heart failure^42^. In Study I, we did not find any correlation between the rapid cortisol and S100A8/A9 responses at 20 min after stress, suggesting that the anti-inflammatory effects of cortisol do not occur fast enough to counteract the rapid S100A8/A9 increase. It is known that cortisol mainly acts at gene and transcription factor level^21^, and thus requires longer time (4–24 h) to reach an adequate effect. Interestingly, in study II we found a significant inverse relationship between the acute cortisol response to stress and the sustained 24 h increase in S100A8/A9 levels in CAD patients, but not in the control group. However, the study population was small and data should be interpreted with caution. It is important to note that the results indicate an association but do not allow us to draw any conclusions about a causal relationship between a dysfunctional HPA axis and S100A8/A9 release in response to stress. The potential inability of a blunted cortisol response to inhibit the pro-inflammatory S100A8/A9 rise will have to be tested in experimental models.

Our study has some important limitations that need mentioning. We were unable to assess whether there were any differences in the rapid S100A8/A9 response to psychological stress between CAD patients and healthy controls, as Study I only included patients. Similarly, we could not conclude whether the participants with a higher early increase in S100A8/A9 also maintained higher S100A8/A9 levels over a 24 h period, since early and late measurements of stress-induced S100A8/A9 were performed in different experimental settings. In the second study, we cannot exclude that the disparities in baseline medication, diastolic blood pressure, heart rate and plasma lipids between patients and controls might have contributed to differences in S100A8/A9 secretion between the two groups. Considering the small number of participants and the large variability in S100A8/A9 response, the differences in S100A8/A9 increase at 24 h after stress between patients and controls have to be interpreted with due caution. Additionally, our study was not powered to detect a prospective link between S100A8/A9 response and increased risk for recurrent coronary events and mortality. Studies in larger cohorts are needed in order to confirm whether there is a significant difference in S100A8/A9 response between patients and controls, and to establish whether the S100A8/A9 response to psychological stress can be used to risk-stratify CAD patients.

In conclusion, we demonstrate for the first time that acute psychological stress triggers systemic release of the pro-inflammatory alarmin S100A8/A9. Our data also indicate that the S100A8/A9 response to stress may be associated with dysregulated cortisol secretion in CAD patients, thereby providing further insight into the link between HPA axis dysfunction, inflammation and CVD.

## Methods

### Study populations

Two cohorts of CAD patients have been used in this study. In Study I, we included 60 CAD patients at 6–12 months after an index cardiac event. The patients had been diagnosed in accordance with the consensus document of the Joint European Society of Cardiology/American College of Cardiology Committee^43^. Fourty-four patients had suffered an acute coronary syndrome [24 non-ST-segment elevation myocardial infarction (NSTEMI), 20 ST-segment-elevation myocardial infarction (STEMI)], and 16 had undergone percutaneous coronary intervention (PCI) due to stable angina. The exclusion criteria were age >75 years, on-going infection, worsened or recurrent angina or readmission to hospital within the previous 10 weeks, moderate to severe heart failure, chronic inflammatory or immunologic disorders, neoplasm, continuous treatment with immunosuppressive/anti-inflammatory agents, drug or alcohol abuse or poor mental function.

In Study II, we included 30 patients at 12–14 weeks after a first-time ACS and 30 healthy controls with equal age and gender distribution randomly selected from the population register. Eleven of the patients had been diagnosed with NSTEMI, and 19 with STEMI. Twenty-eight patients had been treated with thrombolysis and/or PCI, and two had an acute coronary artery by-pass grafting operation (CABG). The controls were all clinically healthy, with neither history nor clinical signs of CAD or other CVD. The exclusion criteria were age >70 years, but otherwise similar to Cohort I. In controls, antihypertensive drugs and statins as primary preventive treatments were allowed. A complete set of blood samples was available from 27 patients and 28 controls in Study II. Written informed consent was obtained from all study participants, the Ethical Review Board of Linköping University approved the research protocol, and the study was conducted according to the ethical guidelines of the Declaration of Helsinki.

### Psychological stress test and sample collection

In both studies, the participants came to the hospital in a fasting state, and were instructed to take prescribed drugs as usual. Testing always started between 07.30–08.00 a.m. Baseline blood pressure and heart rate were recorded with subjects comfortably lying down on a bed. Saliva samples for baseline cortisol were taken before venipuncture and collection of baseline blood samples.

The psychological stress test included two parts with different stressors, an anger recall test and an arithmetic test, with a pause of 2 minutes in-between^16,23^. In the first part, the participant was instructed to recall an event that made him/her angry, frustrated or upset, and to discuss for 6 min what had happened and how that made him/her feel. In the second part, the participants were instructed to count backwards from 700 minus 7 as quickly and accurately as possible, and to reach zero within 4 min. Blood pressure and pulse rate were measured before stress and every 2 min until the end of the test.

In Study I, the second blood sample was collected at 34 min after the start of the first stressor, i.e. 20 min after the completion of the second stressor. In Study II, the second blood sample was collected at 24 h after the stress test. In both cohorts, saliva samples were collected at baseline and at 20 min after stress. For measurement of diurnal cortisol variation, additional saliva samples were collected from Study I participants twice daily on three consecutive days, 30 minutes after awakening and in the evening before going to bed. Saliva collection was performed by placing salivette cotton swabs (Sarstedt, Nürnbrecht, Germany) under the tongue for 2 min. The swabs were then immediately frozen at −20 °C.

### Biochemical analyses

#### S100A8/A9 ELISA

S100A8/A9 was measured by using a previously described ELISA technique^44^. Microtiter plates (Maxisorp, Nunc, Roskilde, Denmark) were coated at 4 °C overnight with 5 μg/ml monoclonal antibody against S100A8/A9 (27E10, BMA Biomedicals AB, August, Switzerland) diluted in PBS pH 7.2, followed by a blocking step using 150 μl of 1% BSA (ICN Biomedicals Inc., Aurora, OH, USA). A buffer containing 0.15M NaCl, 10mM HEPES (Invitrogen, Carlsbad, CA, USA), 1 mM CaCl_2_, 0.02 mM ZnCl_2_, 0.05% Tween 20 and 0.1% BSA was used for dilution of serum samples, detection antibody and streptavidin. Serum samples diluted 1/100 were added to the microtiter plates and incubated for 2h. The wells were thereafter washed three times with PBS containing 0.05% Tween 20, followed by overnight incubation with biotinylated polyclonal antibodies against S100A8/A9 (chicken polyclonal antibody MRP8/14, Abcam, Cambridge, UK) diluted 1/2000. After washing, the antibodies were labeled with ALP-bound streptavidin (Dako, Glostrup, Denmark) diluted 1/1000. Bound streptavidin was visualized with disodium-p-nitrophenyl phosphate (Sigma, St Louis, MO, USA) 1 mg/ml dissolved in 10% (w/v) diethanolamine pH 9.8 containing 50 mM MgCl_2_. The absorbance was measured at 405 nm. All samples were run in duplicate. Optical density values of uncoated wells were used as background and subtracted from the values of the samples. Titration curves obtained from one serum with known concentration were used to calculate the S100A8/A9 concentrations. The lower detection level was 3 ng/mL.

#### Saliva cortisol

Measurement of free cortisol levels in saliva was performed at the accredited Clinical Chemical Laboratory at Karolinska University Hospital, Sweden, using a commercial radioimmunoassay assay, CORT-CT2 (Cisbio, Bioanalyser, Codolet, France), with a limit of detection of 3.0 nmol/L and limit of quantitation of 100 nmol/L. The interassay coefficient of variance was less than 10% according to repeatedly performed quality assessments.

### Statistical analysis

We have used the Student’s t-test to compare the values of normally distributed continuous variables between groups, and the Mann-Whitney U-test for non-normally distributed continuous variables. Differences in categorical variables were assessed with the chi-square test or Fischer’s exact test, as appropriate. Variations in values between different time-points when serial measurements were performed in the same subjects were examined by the Wilcoxon paired test for non-normally distributed variables. Spearman’s rank test and a multivariable linear regression analysis model were used to test associations between percentage S100A8/A9 increase relative to baseline and other clinical and biochemical parameters in the population. Non-normally distributed continuous variables were logarithmated before being used in the linear regression model. A *p*-value under 0.05 was considered to be statistically significant. Values are presented as mean (SD) if normally distributed and median (inter-quartile range) if not normally distributed. All calculations were made using SPSS 22.0 (IBM software, Armonk, NY).

### Data availability statement

The datasets generated during and/or analysed during the current study are available from the corresponding author on reasonable request.
